# RNAs in Brain and Heart Diseases †

**DOI:** 10.3390/ijms21103717

**Published:** 2020-05-25

**Authors:** Dimitris Beis, Inga Zerr, Fabio Martelli, Wolfram Doehner, Yvan Devaux

**Affiliations:** 1 Zebrafish Disease Models lab, Center for Experimental Surgery Clinical and Translational Research, Biomedical Research Foundation Academy of Athens, 11527 Athens, Greece; dbeis@bioacademy.gr; 2 Department of Neurology, Clinical Dementia Centre and DZNE, University Medical School, Georg-August University, 37075 Göttingen, Germany; ingazerr@med.uni-goettingen.de; 3 Molecular Cardiology Laboratory, IRCCS Policlinico San Donato, San Donato Milanese, 20097 Milan, Italy; Fabio.Martelli@grupposandonato.it; 4 Department of Cardiology (Virchow Klinikum), BIH-Center for Regenerative Therapies, and Center for Stroke Research Berlin Charité-Universitätsmedizin, 13353 Berlin, Germany; wolfram.doehner@charite.de; 5 Cardiovascular Research Unit, Department of Population Health, Luxembourg Institute of Health, L-1445 Strassen, Luxembourg

**Keywords:** brain, heart, comorbidities, noncoding RNAs, neurocardiology

## Abstract

In the era of single-cell analysis, one always has to keep in mind the systemic nature of various diseases and how these diseases could be optimally studied. Comorbidities of the heart in neurological diseases as well as of the brain in cardiovascular diseases are prevalent, but how interactions in the brain–heart axis affect disease development and progression has been poorly addressed. Several brain and heart diseases share common risk factors. A better understanding of the brain–heart interactions will provide better insights for future treatment and personalization of healthcare, for heart failure patients’ benefit notably. We review here emerging evidence that studying noncoding RNAs in the brain–heart axis could be pivotal in understanding these interactions. We also introduce the Special Issue of the International Journal of Molecular Sciences RNAs in Brain and Heart Diseases—EU-CardioRNA COST Action.

## 1. Introduction

The concept of “Neurocardiology”, although known since the early 1980s [1], is re-emerging. Epidemiological data link psychosocial stress to cardiovascular disease [2]. Low socioeconomic status induces stress to the brain through chronic activation of the amygdala, leading to activation of inflammation, enhanced atherogenesis, and cardiovascular events [3].

## 2. Physiological Interactions between the Heart and Brain

Neurodegenerative diseases share risk factors with cardiovascular diseases, such as age, high blood pressure, sex, high cholesterol, and presence of amyloid oligomers [4]. Mutations in the β-secretase-1 gene lead to β-amyloid peptide accumulation and chronic inflammation [4,5]. Alzheimer’s disease (AD) patients show diastolic dysfunction and other cardiac functional defects reminiscent of cardiac amyloidosis [6,7]. On the other side, ischemic heart failure patients display increased left-ventricular levels of both β-secretase-1 (BACE1) and β-amyloid peptides, as well as the noncoding RNA antisense transcript BACE1-AS [8], suggesting common pathogenetic mechanisms with Alzheimer’s disease. Moreover, increased β-amyloid plasma levels allow identifying patients at high risk for cardiovascular death [9]. Therefore, compromised myocardial function and intramyocardial deposits of Aβ have been reported in AD patients and suggestions are made that AD may be viewed rather as a systemic disease [6,7,8,9]. Parkinson’s disease (PD) also affects cardiac function through mainly damage to the sympathetic nervous system and the cardiac autonomic system, which control heart rate and blood pressure. However, the cardiovascular aspect of PD (as well as of the related neurodegenerative α-synucleinopathies) is often disregarded, which might account for the sudden unexpected deaths of these patients [10].

**Figure 1:**
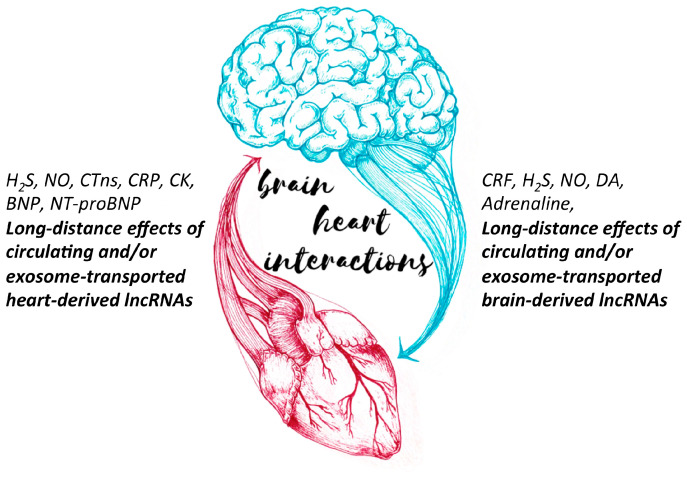
Two-way traffic of signaling molecules and noncoding RNAs drives the brain–heart interactions.

Cardiac arrhythmias are considered to be the leading cause of stroke, while the central nervous system controls cardiovascular function through the sympathetic and parasympathetic systems. A recent study by Templin and colleagues suggests that hypoconnectivity in central brain regions may contribute to the development of left ventricular dysfunction in patients with Takotsubo syndrome [11]. Hydrogen sulfide and nitric oxide are gasotransmitters produced by the brain and the heart and have the potential to act locally as well as remotely through stimulation of angiogenesis, neovascularization, and postischemic cardiac repair [12]. Several circulating molecules have been identified to mediate signals from the brain to the heart. These involve the hypothalamic–pituitary–adrenal axis and the corticosteroid releasing factor [13], as well as catecholamines [14] that act on heart physiology. Following a stroke or other brain injuries, several circulating biomarkers are elevated and have some potential as indicators of organ dysfunction and predictors of adverse prognosis. These include creatine kinase, N-terminal pro-brain natriuretic peptide, C-reactive protein, and cardiac troponins [15]. Damage in the dopaminergic system and disturbances of dopamine levels affect both the central nervous system (CNS) as well as the periphery, including the heart and the pancreas, reviewed in [16]. In patients resuscitated from cardiac arrest, higher heartbeat-evoked brain potentials, as recorded by electroencephalographic combined with electrocardiographic assessment, are associated with a higher survival at six months [17]. However, adrenaline administration to restart the heart was associated with more severe neurological problems [18]. Both cardiac damage and brain injury elicit an initially local and ultimately systemic inflammatory response that affects multiple organs. This response is orchestrated by signaling molecules such as tumor necrosis factor alpha, interleukins, and other proinflammatory cytokines. These observations support the importance of taking into account potential brain–heart communications when studying and treating brain or cardiac diseases (Figure 1).

## 3. Noncoding RNAs as Potential Long-Distance Signal Mediators and Circulating Biomarkers

Noncoding RNAs are a relatively recently described but significant subpopulation of the transcriptome that emerged following the latest developments in next-generation sequencing technologies. A large proportion of these molecules are tissue- and developmental-stage-specific, as well as dysregulated in diseases [19]. Noncoding RNAs include populations of microRNAs, transfer RNAs, ribosomal RNAs, small nuclear RNAs, and others. Long noncoding RNAs (LncRNAs) are the subclass with transcripts longer than 200 nucleotides and circular RNAs are the products of backsplicing events. Exosomes are known to contain different types of RNA, among which small-sized microRNAs play a preponderant role [20]. LncRNAs and circular RNAs are present in exosomes, although their paracrine function remains uncertain. RNA molecules packed in extracellular vesicles or exosomes can be actively released from cells, inflammatory or others. It appears that this paracrine process is highly regulated, regarding the cargo load and the target tissues. However, it can also be tailored according to demand, enabling the efficient delivery of therapeutic compounds, manipulating the microenvironment of the desired tissue.

The blood–brain barrier is disrupted after an ischemic insult [15], which allows the release of brain mediators to the blood circulation, such as neuron-specific enolase, which is used as an indicator of the extent of brain damage to predict neurological outcome in cardiac arrest patients [21]. Not only metabolites and proteins but also microRNAs appear to be able to cross the blood–brain barrier after ischemia. Indeed, blood levels of the brain-enriched miR-124-3p increase after cardiac arrest and predict neurological outcome and survival [22]. Other noncoding RNAs may aid in outcome prediction after cardiac arrest [23]. LncRNAs and other protein-coding RNAs are regulated in the cerebral cortex of rats after return of spontaneous circulation following electrical shock-mediated cardiac arrest [24].

Noncoding RNAs have the potential to regulate gene expression at multiple epigenetics levels and affect heart failure development and progression [25,26,27].

They regulate various microenvironment factors that can participate in restricting the damaged area as well as switching from a fibrotic to a regenerative response to acute stress. It is becoming, therefore, very appealing to consider that noncoding RNAs could mediate brain–heart communication and affect neurological damage, cardiac dysfunction, and repair, and might functionally contribute to the clinical outcome following both cerebral and cardiac events. Noncoding RNAs linking the brain and the heart may thus constitute a novel reservoir of biomarkers and therapeutic targets.

## 4. Perspectives and Outlook

However, before considering translating noncoding RNAs to the clinic, much remains to be done:

- Perform high-throughput-based identification of the RNAs with potential paracrine roles between the brain and the heart in different disease conditions;
- Demonstrate that candidate RNA biomarkers cross the blood–brain barrier;
- Address the paracrine role of RNAs using in vivo and in vitro approaches;
- Identify and validate in patient cohorts brain- or heart-specific RNA biomarkers associated with clinical outcome.

The success of the above will largely rely on:

- Resolving technical challenges inherent to the identification of candidate RNAs and their use as biomarkers and therapeutic targets;
- Designing optimal protocols for biological sample collection, storage, and processing (i.e., RNA measurement);
- Developing unbiased and corrected for multiple testing bioinformatics and biostatistics approaches for RNA biomarker discovery;
- Using existing or de novo multicenter patient cohorts for independent and properly sized validation of RNA candidates identified in discovery phases using next-generation sequencing or other high-throughput techniques;
- Addressing sex differences;
- Joining complementary forces and expertise of clinicians, researchers, information technology, and biostatistics specialists to build collaborative research projects addressing the brain–heart axis in a systems-based manner.

The EU-CardioRNA COST Action CA17129 (www.cardiorna.eu) aims to build such translational research programs through stimulating networking activities on RNAs in the brain and heart fields [26,28].

**Figure 2:**
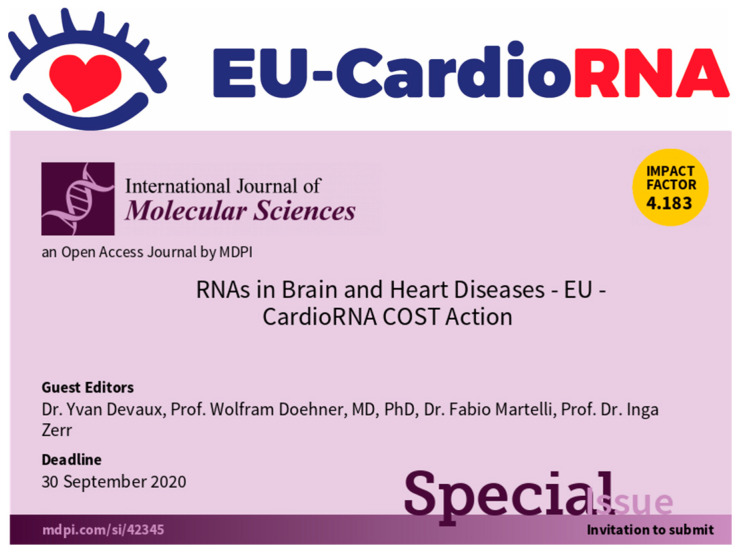
Banner of Special Issue “RNAs in Brain and Heart Diseases - EU-CardioRNA COST Action”.

Through the Special Issue “RNAs in Brain and Heart Diseases” in International Journal of Molecular Sciences (https://www.mdpi.com/journal/ijms/special_issues/rna_brain_heart_cost), members of the EU-CardioRNA COST Action CA17129 aim to provide a forum for reporting and discussion on the topic of RNAs in brain and heart diseases, focusing on organ interactions and common mechanisms between diseases. The banner is showed as Figure 2. The Special Issue specially welcomes regular research articles or review articles focusing on brain disease affecting the cardiovascular system or heart disease affecting the neurological system. 

In summary, recent studies support the existence of physiological and functional links between the brain and the heart. Successful identification of such links would allow a better evaluation of the damage in either organ, as well as a more accurate prognostication of patients, fostering the implementation of personalized medicine. The degree by which RNAs mediate interactions in the brain–heart axis and how they affect heart failure progression remain understudied. Multidisciplinary research consortia are required to clarify this possibility and push the emerging field of Neurocardiology forward.

## Abbreviations

COST	Cooperation in Science and Technology
ncRNAs	Noncoding RNAs
LncRNAs	Long noncoding RNAs
BACE1	β-secretase-1
AD	Alzheimer’s disease
PD	Parkinson’s disease
